# Genome Sequence of Rapid Beer-Spoiling Isolate *Lactobacillus brevis* BSO 464

**DOI:** 10.1128/genomeA.01411-15

**Published:** 2015-12-03

**Authors:** Jordyn Bergsveinson, Vanessa Pittet, Emily Ewen, Nina Baecker, Barry Ziola

**Affiliations:** Department of Pathology and Laboratory Medicine, College of Medicine, University of Saskatchewan, Saskatoon, Saskatchewan, Canada

## Abstract

The genome of brewery-isolate *Lactobacillus brevis* BSO 464 was sequenced and assembly produced a chromosome and eight plasmids. This bacterium tolerates dissolved CO_2_/pressure and can rapidly spoil packaged beer. This genome is useful for analyzing the genetics associated with beer spoilage by lactic acid bacteria.

## GENOME ANNOUNCEMENT

Brewery isolate *Lactobacillus brevis* BSO 464 (Lb464; obtained from the Brewing Research Foundation, Oxoid, UK) can resist the headspace pressure and dissolved CO_2_ in packaged beer, making it a virulent beer-spoiler (1–3). Lb464 was grown overnight in MRS broth culture at 30°C and DNA was extracted with the UltraClean Microbial DNA Isolation Kit (Mo-Bio Laboratories, Carlsbad, CA). Heating at 10 min at 70°C was used prior to bead-beating to optimize cell lysis.

Sequencing was performed using the Roche 454 FLX platform at the National Research Council Plant Biotechnology Institute (NRC PBI; Saskatoon, SK). Two separate runs yielded 330,771 unpaired and 567,735 paired reads for ~30× coverage. Reads were assembled using Newbler GS *De Novo* assembler v2.5.3. Hawkeye (4) was used to visualize the 236 contigs across 17 scaffolds to identify contigs that could be potentially joined by sequencing of PCR amplicons (via the NRC PBI ABI 3700*xl* platform).

The Lb464 genome was assembled into a 2,503,991-bp circular chromosome (G+C content 45.7%) that had 8,461 bp cumulative of gaps due to transposase or repetitive regions not allowing PCR-based sequencing. Additionally, eight plasmids were assembled (G+C content ranged from 39.1% to 42.4%): pLb464-1 (15,324 bp), pLb464-2 (28,459 bp), pLb464-3 (22,411 bp), pLb464-4 (84,941 bp), pLb464-5 (10,867 bp), pLb464-6 (5018 bp), Lb464-7 (2353 bp), and pLb464-8 (49,835 bp, with a 1,000 bp gap due to repetitive regions) (2). Among sequenced lactobacilli, Lb464 has the second highest known plasmid number after *L. brevis* KB290 (KB290; GCA_000359625.1) with nine plasmids (5). Mapping the Lb464 chromosome via DNA Plotter (6) revealed an atypical G+C-skew that has no symmetric bias in nucleotide composition of leading and lagging DNA strands relative to the replication origin (7). In Lb464, the AT-rich lagging strand encroaches into the normally G+C-rich leading strand by ~500 Kb. This could indicate a potential misassembly (inversion) of a ~500 Kb section of the Lb464 chromosome. However, the Lb464 G+C-skew is believed real due to similarity with the atypical chromosomal G+C-skew found in *L. brevis* ATCC 367^T^ (Lb367; CP00416.1).

Lb464 genome annotation by the NCBI PGAP pathway (8) produced 2,615 coding sequences, 6 rRNA operons, and 48 tRNAs (tRNAs for cysteine, histidine, and tryptophan are absent). One chromosomal clustered regularly interspaced short palindromic repeat (CRISPR) region is present, and 79 transposase genes of the ISL3 and IS30 family are found within the Lb464 genome, which is more than in Lb367 and KB290. Lb464 codes for several of the transcripts shown to be important in beer spoilage in a transcriptional study with *Pediococcus claussenii* (9), notably agmatine deiminase, putrescine carbamoyltransferase, and transporters malate, citrate, and other carbohydrate and nitrogen sources. Lb464 also contains the previously identified hop-tolerance genes *hitA* (10), *horA* (11), and *horC* (12) on pLb464-3, pLb464-1, and pLb464-2, respectively. However, only pLb464-2, pLb464-4, and pLb464-8, have been shown to be important for Lb464 growth in beer (2). Based on the PGAP annotation, these three plasmids code for 190 other proteins, including 106 hypothetical proteins, all of which need to be investigated for their contribution to the Lb464 beer-spoilage phenotype.

### Nucleotide sequence accession numbers.

The sequences for *Lactobacillus brevis* BSO 464 were deposited in GenBank under accession numbers CP005977, CP005978, CP005979, CP005980, CP005981, CP005982, CP005983, CP005984, and CP005985 for the chromosome and plasmids pLb464-1 to pLb464-8, respectively.
